# Absence of PKC-Alpha Attenuates Lithium-Induced Nephrogenic Diabetes Insipidus

**DOI:** 10.1371/journal.pone.0101753

**Published:** 2014-07-09

**Authors:** Jae H. Sim, Nathaniel J. Himmel, Sara K. Redd, Fadi E. Pulous, Richard T. Rogers, Lauren N. Black, Seongun M. Hong, Tobias N. von Bergen, Mitsi A. Blount

**Affiliations:** 1 Renal Division, Department of Medicine, Emory University School of Medicine, Atlanta, Georgia, United States of America; 2 Department of Physiology, Emory University School of Medicine, Atlanta, Georgia, United States of America; University of Geneva, Switzerland

## Abstract

Lithium, an effective antipsychotic, induces nephrogenic diabetes insipidus (NDI) in ∼40% of patients. The decreased capacity to concentrate urine is likely due to lithium acutely disrupting the cAMP pathway and chronically reducing urea transporter (UT-A1) and water channel (AQP2) expression in the inner medulla. Targeting an alternative signaling pathway, such as PKC-mediated signaling, may be an effective method of treating lithium-induced polyuria. PKC-alpha null mice (PKCα KO) and strain-matched wild type (WT) controls were treated with lithium for 0, 3 or 5 days. WT mice had increased urine output and lowered urine osmolality after 3 and 5 days of treatment whereas PKCα KO mice had no change in urine output or concentration. Western blot analysis revealed that AQP2 expression in medullary tissues was lowered after 3 and 5 days in WT mice; however, AQP2 was unchanged in PKCα KO. Similar results were observed with UT-A1 expression. Animals were also treated with lithium for 6 weeks. Lithium-treated WT mice had 19-fold increased urine output whereas treated PKCα KO animals had a 4-fold increase in output. AQP2 and UT-A1 expression was lowered in 6 week lithium-treated WT animals whereas in treated PKCα KO mice, AQP2 was only reduced by 2-fold and UT-A1 expression was unaffected. Urinary sodium, potassium and calcium were elevated in lithium-fed WT but not in lithium-fed PKCα KO mice. Our data show that ablation of PKCα preserves AQP2 and UT-A1 protein expression and localization in lithium-induced NDI, and prevents the development of the severe polyuria associated with lithium therapy.

## Introduction

Although lithium is an older antipsychotic, it still remains the most common treatment for bipolar disorder [1]. Lithium also has beneficial effects in multiple other CNS disorders including stroke, multiple sclerosis, HIV-associated neurotoxicity and Huntington disease [2]. Although lithium is effective at treating these and other CNS disorders, the drug is also known to be associated with renal, neurological and endocrine side effects [1]. One of the renal side effects associated with lithium therapy is nephrogenic diabetes insipidus (NDI) which presents in approximately 40% of patients [3], [4]. Affected patients present with polyuria, polydipsia, reduced capacity to produce concentrated urine and an inability to respond to vasopressin [3], [4]. The advancing polyuria associated with lithium-acquired NDI may appear early in the treatment regimen and can be considered a contraindication to continued use. In many cases with chronic use, lithium-induced NDI cannot be reversed thus discontinuing therapy after a certain point may not be advantageous in reducing this side effect [5]. Ensuing NDI is particularly problematic as the risk for acute renal failure is significantly elevated due to increased circumstance for acute lithium toxicity and/or dehydration from severe polydipsia/polyuria.

Although not all of the signaling mechanisms underlying lithium-induced NDI have been elucidated, acute administration of lithium inhibits the formation of cAMP [6]–[8]. Cyclic AMP-dependent phosphorylation of two critical transporters in the urine concentration mechanism, aquaporin-2 (AQP2) and the urea transporter, UT-A1, is required for translocation and insertion of these transporters into the apical plasma membrane of the inner medullary collecting duct (IMCD) [9], [10]. This initial dysregulation of vasopressin-regulated water reabsorption contributes to the urine-concentrating defect; however, long-term lithium treatment decreases the protein abundance AQP2 and UT-A1 exacerbating the effect [11]. Although lithium-dampened cAMP production is likely the primary cause of NDI, lithium dysregulation of renal prostaglandins [12], altered purinergic signaling [13] and modifications of the phosphatidylinositol signaling pathway [14]–[18] have also been implicated.

To prevent advancing renal side effects resulting from lithium therapy, physicians may have to remove the patient from treatment regardless of its effectiveness on psychotic episodes. With the increasing popularity of lithium for treatment of other CNS disorders, there is an increased need to alleviate potential renal side effects that may result in early termination of an otherwise effective treatment. Recent studies revealing cAMP-independent pathways that regulate urine concentration [17], [19], [20] indicate that targeting regulatory proteins in these signaling cascades may provide novel pharmacological targets to treat vasopressin insensitive NDI. Recently, PKCα, a kinase involved in phosphatidylinositol signaling, has been shown to regulate AQP2 and UT-A1 function independently from cAMP [16]–[18]. We explored the idea that in the absence of PKCα, the severity of lithium-mediated NDI would be affected using a PKCα null mouse model with the ultimate goal of identifying a potential therapeutic site for prevention of this renal side effect of lithium therapy. The findings of our study show that ablation of PKCα significantly offsets lithium-induced NDI in part by preserving the transporters involved in the urine concentration mechanism.

## Methods

### Animals

All animal protocols were approved by the Emory University Institutional Animal Care and Use Committee. PKCα^−/−^ mice were initially obtained from Dr. Jeffery Molkentin (Cincinnati Children's Hospital Medical Center) [21]. The mice were bred in parallel with wild-type mice from a mixed C57BL/6 x 129 genetic background (Jackson Laboratory, Bar Harbor, ME). Each three to four generations PKCα^−/−^ and PKCα^+/+^ were crossed to generate heterozygotes, which were then bred to produce wild-type (WT) and PKCα-null (PKCα KO) litermates, which were then bred separately. All mice were male, ranged between 7–9 weeks of age and between 23-25 grams at the beginning of lithium treatment. For short-term lithium treatment, PKCα KO and WT mice were injected intraperitoneally with 40 mmol/kg LiCl in saline every 24 hours for 3 or 5 days. For long-term treatment studies, PKCα KO and WT mice were fed either standard diet (containing 23% protein) supplemented with LiCl (40 mmol/kg; Harlan Teklad, Madison, WI) or standard diet without supplementation for 6 weeks. In both treatment groups, mice were given free access to tap water and a salt block to maintain sodium balance and prevent lithium intoxication. Mice were individually housed in a Tecniplast Single Mouse Metabolic Cage, (Tecniplast USA Inc, Exton, PA) for a 24-hour acclimation period followed by an additional 24 hours to measure food and fluid intake and collect urine for analysis.

### Western Blot Analysis

Proteins (20 µg/lane) were size-separated by SDS-PAGE on 10% gels and then electroblotted to polyvinylidene difluoride membranes (Immobilon, Millipore, Bedford, MA). After being blocked with 5% nonfat dry milk for 1 hour, blots were incubated with primary antibody overnight at 4°C. Specifically, either a 1∶2000 dilution of an antibody generated against the C-terminal domain of AQP2 (purchased from StressMarq (Victoria, BC, Canada)), or a 1∶1000 dilution of an antibody generated against the C-terminal domain of the UT-A1 transporter (fully characterized by our source of the antibody, Dr. Jeff M. Sands [22]) was used for AQP2 and UT-A1 detection, respectively. Blots were next washed three times in tris-buffered saline with 0.5% Tween 20 and then incubated for 2 hours with Alexa Fluor 680-linked anti-rabbit IgG (1∶4000; Molecular Probes, Eugene, OR). Bound secondary antibody was visualized using infrared detection with the LI-COR Odyssey protein analysis system (Lincoln, NE) and densitometry of the desired band was collected. To insure equal loading and quantify our densitometric scanning, blots were also probed for β tubulin (1∶500; Thermo Fisher Scientific, Waltham, MA) as a loading control. Positive detection was observed following incubation with the corresponding secondary antibody, DyLight 800 conjugated anti-mouse IgG (1∶5000; Cell Signaling Technology, Danvers, MA). The Odyssey system was used to collect densitometry of the β tubulin band. Results are expressed as arbitrary units normalized to the densitometry of the loading control, β tubulin.

### Analysis of Urine and Serum Samples

Urine osmolality was measured on a Wescor 5520 Vapor Pressure Osmometer (Wescor, Logan, UT). Serum lithium levels and urinary sodium, chloride and potassium were measured by EasyLyte (Medica, Bedford, MA) instrument. Urine creatinine was determined by the Jaffe reaction and serum creatinine was measured by high-performance liquid chromatography. Urinary calcium was measured by atomic absorption and urinary protein by the Lowery method.

### Immunohistochemistry

Kidneys from all four experimental groups were perfusion fixed with 4% paraformaldehyde. Paraffin sections (5 µm thick) were cut on a Leica microtome and dried overnight at 37°C. Slides were then placed in xylene overnight and then rehydrated in a graded series of ethanol (30 min in 99% ethanol and 10 min in 96% ethanol) and endogenous peroxidases were blocked with H_2_O_2_ (1% H_2_O_2_ solution in methanol) for 30 minutes. Target retrieval was accomplished by microwaving samples with 10 mmol/l Tris and 0.5 mmol/l EGTA (TEG) buffer. To prevent nonspecific antibody binding, sections were further blocked with 50 mmol/l NH_4_Cl in PBS (30 min). Tissue sections were incubated overnight at 4°C in a humidified chamber with primary antibody (1∶50,000 C-terminal UT-A1; 1∶10,000 C-terminal AQP2). The tissue sections were then incubated the following day with secondary antibody (anti-rabbit IgG) conjugated to horseradish peroxidase (1∶200, GE Healthcare/Amersham Biosciences, Piscataway, NJ) for 2 hours at room temperature. Positive staining was visualized using a diaminobenzidine dye (brown; Amreso, Solon, OH). Cell nuclei were also counterstained with hematoxylin (blue; EMD Chemicals, Gibbstown, NJ). Images were collected with an Olympus 1×51 inverted microscope using a SIS-CC12 CLR camera.

### Statistical analysis

Quantitative data are expressed as mean ± SEM. Differences were determined by either t-test or the Kruskal–Wallis one-way analysis of variance where appropriate using GraphPad Instat Software (La Jolla, CA).

## Results

### Water homeostasis in short-term lithium treatment

**Figure 1:**
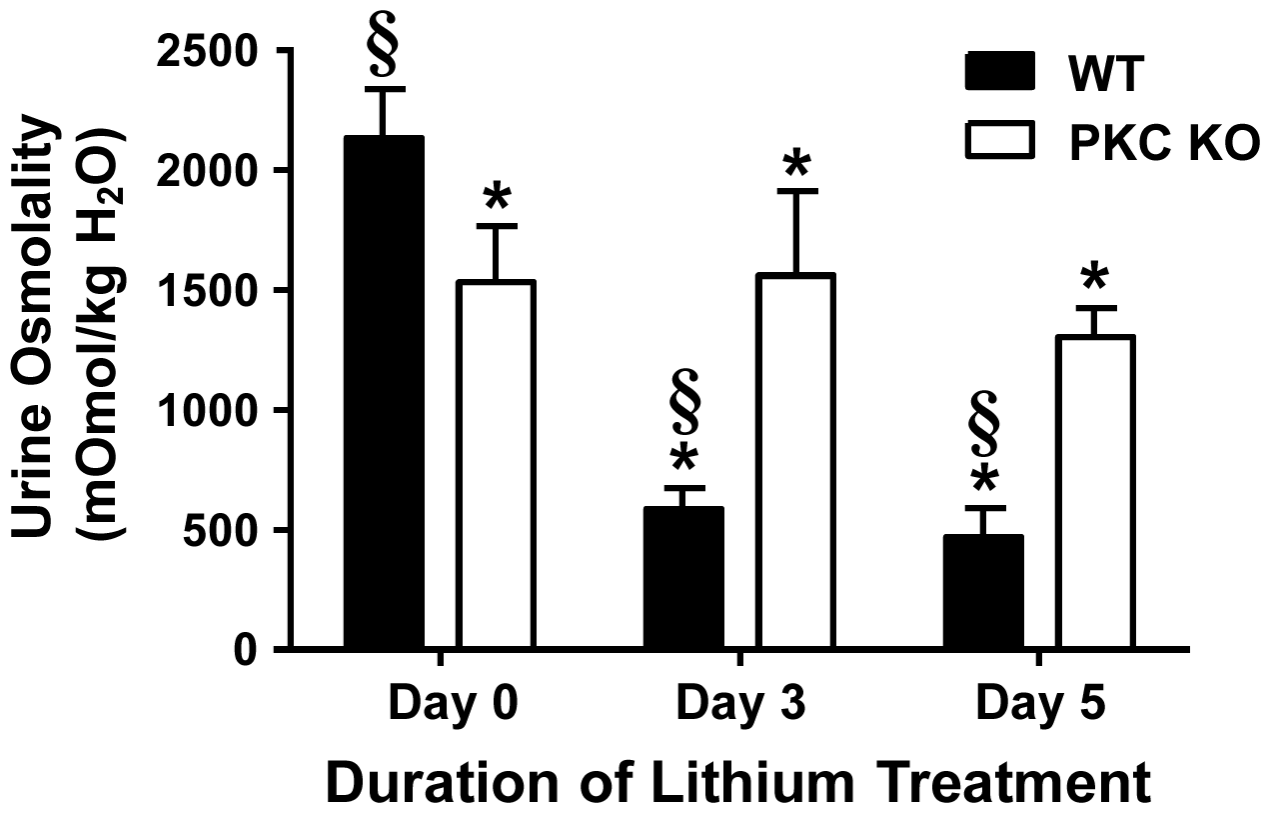
PKCα KO mice do not develop polyuria in response to short-term lithium treatment. PKCα KO mice and littermate controls (WT) were injected daily with 40 mmol/kg of lithium for 3 or 5 consecutive days. Urine was collected via metabolic cages and urine osmolality was determined. Data are presented as mean ± SEM where *  =  p<0.05 vs. WT day 0 and §  =  p<0.05 vs. PKCα KO day 0 is deemed significant. *n = 6*.

We and others have demonstrated that rodents become polyuric within days of lithium treatment [4], [12], [23]–[25]. Similarly, wild-type littermates (WT) generated 5.3±0.3 ml/24 h urine in as little as 3 days following daily lithium injections (40 mmol/kg/d) (Table 1). This urine was 4-fold more dilute than that collected from untreated, WT mice (Figure 1). Following 5 days of lithium injections, WT mice produced 7.4±0.5 ml/24 h urine that remained significantly dilute (Figure 1). Mice with a global deletion of PKCα (PKCα KO) did not generate more urine after 3 or 5 days of lithium-treatment and osmolality was not significantly altered (Figure 1); however, our data confirm previous reports of slight but significant concentrating defect in PKCα KO mice [18] (Table 1). Serum lithium remained below toxicity levels throughout the acute experiments and kidney function was preserved as evidenced by the stable creatinine clearance and urinary protein:urinary creatinine ratio values (Table 1). We conclude that although lithium can induce polyuria within 3 days of treatment, the absence of PKCα protected mice from generating large amounts of dilute urine in the same time frame.

**Table 1:** Acute lithium treatment does not induce NDI in PKCα KO mice.

	Urine Output (mL/24 h)	Urine Osmolality (mOsm/kg)	Serum Lithium (mM/L)	Uprot/Uosm ratio (mg/L/mOsm/kg)	CrCl/BW (µL/min/g)
**WT, Day 0**	1.8±0.4^§^	2131±205^§^	<0.2	0.13±0.01	3.5±1.8
**WT, Day 3**	5.3±0.3*^§^	593.5±52.3*^§^	0.54±0.1*^§^	0.10±0.02	3.8±1.4
**WT, Day 5**	7.4±0.5*^§^	468.6±88.8*^§^	0.71±0.1*^§^	0.12±0.01	3.7±1.7
**KO, Day 0**	2.3±0.5*	1530±235*	<0.2	0.09±0.01	3.9±1.6
**KO, Day 3**	1.9±0.3*	1559±351*	0.64±0.1*^§^	0.10±0.03	4.1±1.5
**KO, Day 5**	2.1±0.3*	1402±122*	0.78±0.2*^§^	0.08±0.02	3.8±1.8

PKCα KO and WT mice were injected intraperitoneally with 40 mmol/kg LiCl in saline every 24 hours up to 3 or 5 days. Single animals were subsequently placed in individual metabolic cages and urine and serum were collected after 0, 3 or 5 days of daily lithium treatments. Uprot/Uosm  =  urinary protein/urine osmolality ratio, CrCl/BW  =  creatinine clearance normalized to body weight (23–25 g). Data are presented as mean ± SEM where *  =  p<0.05 vs. WT day 0 and §  =  p<0.05 vs. PKCα KO day 0 is deemed significantly different. *n* = 6.

### Aquaporin and urea transporter channel expression in short-term lithium treatment

**Figure 2:**
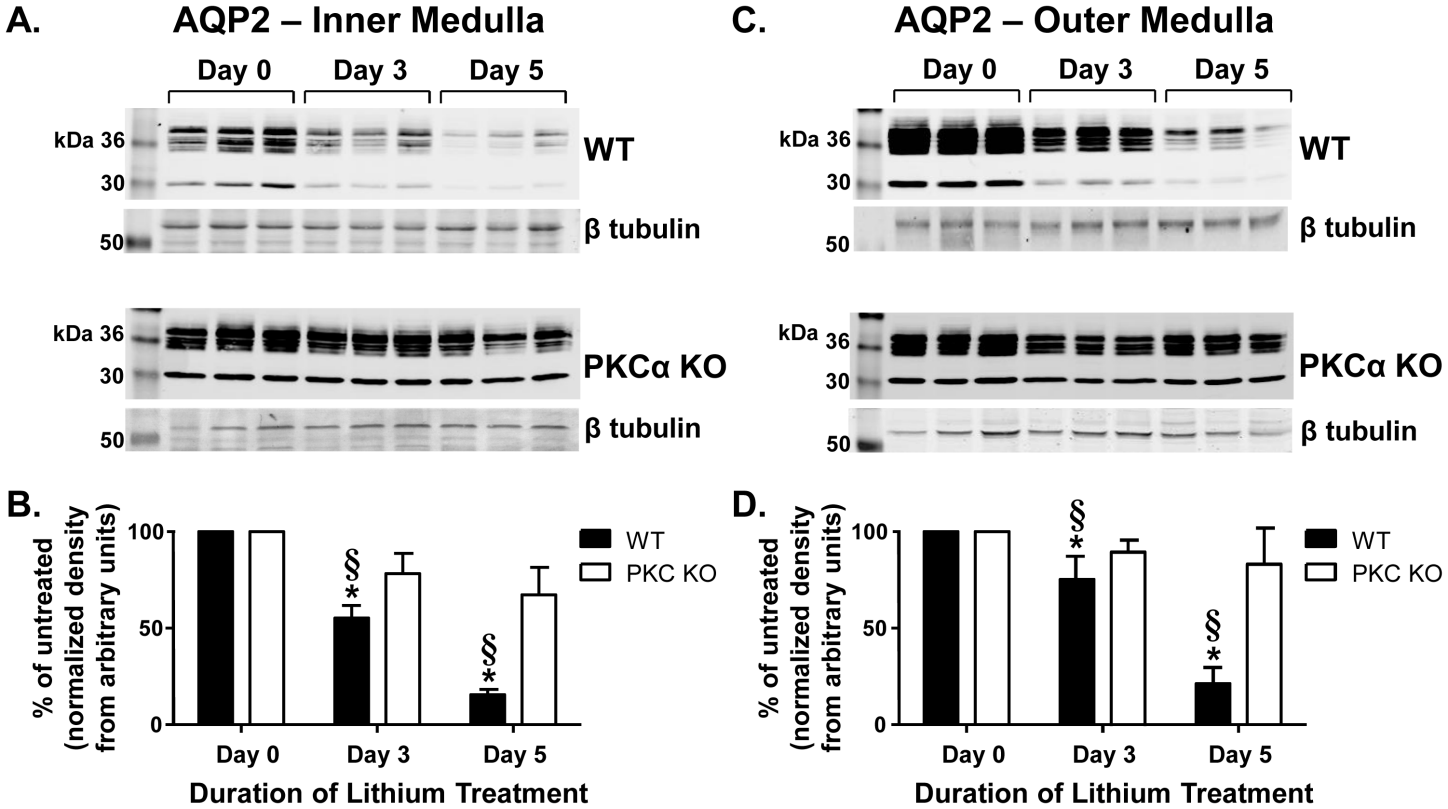
AQP2 expression is not changed in short-term lithium-treated PKCα KO mice. A) IM tissue collected from injected WT and PKCα KO mice was subjected to Western blot analysis and probed for AQP2. Representative blots showing both nonglycosylated (29-kDa) and glycosylated (35- to 50-kDa) AQP2 [59], and the corresponding loading control, β tubulin. Each lane represents one animal. B) Combined densitometry of all forms of AQP2 in the IM normalized to β tubulin. C) OM tissue probed for AQP2 and β tubulin. D) Combined densitometry of all forms of AQP2 in the OM normalized to β tubulin. Data are presented as percent difference in expression from Day 0, untreated mice as mean ± SEM where *  =  p<0.05 vs. WT day 0 and §  =  p<0.05 vs. PKCα KO day 0 is deemed significant. *n = 6*.

Two transporters located in the apical membrane of the IMCD that are responsible for urine concentration are the water channel, AQP2, and the urea transporter, UT-A1 [26], [27]. Absence or dampened expression of one or both of these transporters frequently occurs with clinical maladies that present with polyuria including NDI [28]. In WT mice, combined glycosylated and unglycosylated AQP2 expression in the inner medulla was decreased ∼45% following 3 days of lithium injections and ∼84% with continued treatment for 5 days (Figure 2B). PKCα KO mice did not display a reduction of AQP2 abundance in the inner medulla after 3 or 5 days of lithium treatment (Figure 2A). We observed a similar pattern in lithium-induced reduction of AQP2 expression in outer medullary tissues from WT mice but not from PKCα KO (Figure 2C).

**Figure 3:**
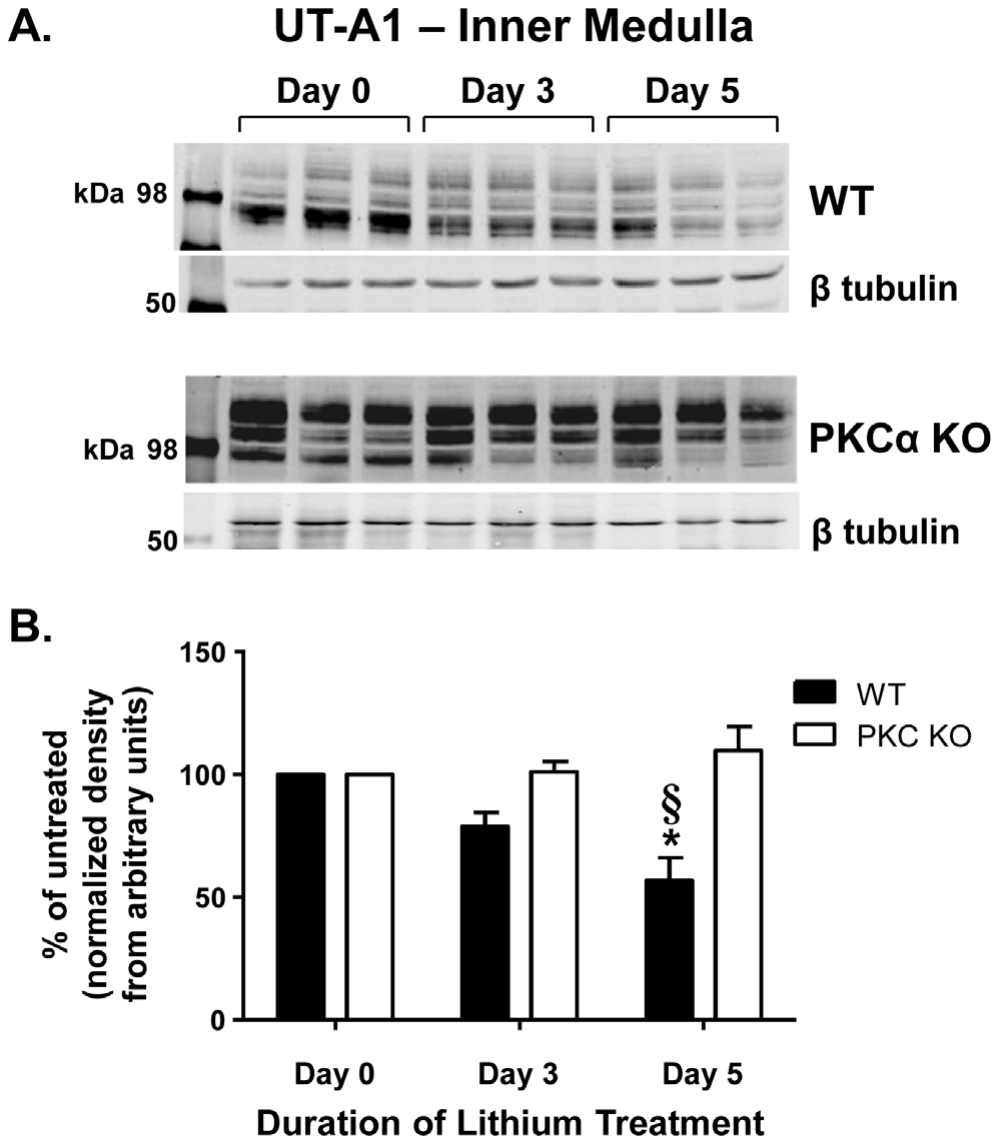
UT-A1 expression is not changed in short-term lithium-treated PKCα KO mice. IM tissue collected from injected WT and PKCα KO mice was subjected to Western blot analysis and probed for UT-A1. A) Representative blots showing the multiple glycosylated forms of UT-A1 that span between 97 and 117 kDa [60], and the corresponding loading control, β tubulin. Each lane represents one animal. B) Combined densitometry of all glycosylated forms of UT-A1 normalized to β tubulin. Data are presented as percent difference in expression from Day 0, untreated mice as mean ± SEM where *  =  p<0.05 vs. WT day 0 and §  =  p<0.05 vs. PKCα KO day 0 is deemed significant. *n = 6*.

We also observed that total glycosylated UT-A1 expression was reduced ∼43% in the inner medulla of WT mice after 5 days of lithium injections; however, UT-A1 abundance was unaffected by lithium treatment in the PKCα KO mice (Figure 3). We did not investigate alterations of UT-A1 expression in the outer medulla because of the transporters exclusive location to the renal papilla [29].

### Water homeostasis and kidney function in long-term lithium treatment

To determine if the absence of PKCα prevents lithium-induced NDI for an extended time, WT and PKCα KO mice were fed either standard chow or chow containing 40 mmol/kg lithium ad libitum for 6 weeks. After 6 weeks, lithium-treated mice had elevated lithium serum levels (Table 2), comparable to therapeutic levels in humans [30]. Mice from all four experimental groups were housed in metabolic cages during the final 72 hours of treatment. Following 24 hours of metabolic cage acclimation, we measured food and water intake and collected urine for 24 hours. There was no difference in food intake among the treated and untreated animals (data not shown); however, lithium treatment increased water intake in both WT and PKCα KO mice albeit polydipsia was increased 10-fold in lithium-treated WT mice and only 3-fold in lithium-treated PKCα KO mice (Table 2). Urine output was also significantly elevated in lithium-treated PKCα KO mice but not to the extent as observed in lithium-treated WT mice (Table 2). Lithium decreased urine osmolality corroborating the developing polyuria with treatment. We found that lithium did not change the creatinine clearance rate in either the WT or PKCα KO mice indicating that 6-week lithium treatment did not alter kidney function in these animals. Furthermore, the urinary protein-osmolality ratio was not significantly different among the four experimental groups confirming the absence of proteinuria [31] (Table 2).

**Table 2:** Lithium-induced NDI is attenuated in PKCα KO mice.

	WT		PKCα KO	
	0 WK	6 WK	0 WK	6 WK
**Water Intake** (mL/24 h)	3.6±0.6	30±3.8*^§^	4.3±0.5	12±1.4*^§^
**Urine Output** (mL/24 h)	1.9±0.3^§^	22.4±3.5*^§^	2.7±0.5*	7.5±0.8*^§^
**Urine Osmolality** (mOsm/kg)	2133±201^§^	477±151*^§^	1430±100*	808±47.9*^§^
**Serum Lithium** (mM/L)	<0.2	0.74±0.12*^§^	<0.2	0.82±0.04*^§^
**Uprot/Uosm ratio** (mg/L/mOsm/kg)	0.12±0.03	0.10±0.02	0.08±0.01	0.07±0.02
**CrCl/BW** (µL/min/g)	3.4±1.6	3.9±1.2	4.1±1.7	4.4±1.8

WT and PKCα KO mice were provided standard chow or chow containing lithium (40 mmol/kg) for 6 weeks. Single animals were subsequently placed in individual metabolic cages to determine 24-h water intake. Urine and serum were also collected at this time point for metabolic determinations. Uprot/Uosm  =  urinary protein/urine osmolality ratio, CrCl/BW  =  creatinine clearance normalized to body weight (29–30 g). Data are presented as mean ± SEM where *  =  p<0.05 vs. WT day 0 and §  =  p<0.05 vs. PKCα KO day 0 is deemed significantly different. *n = 5*.

### Aquaporin and urea transporter channel expression and localization in long-term lithium treatment

**Figure 4:**
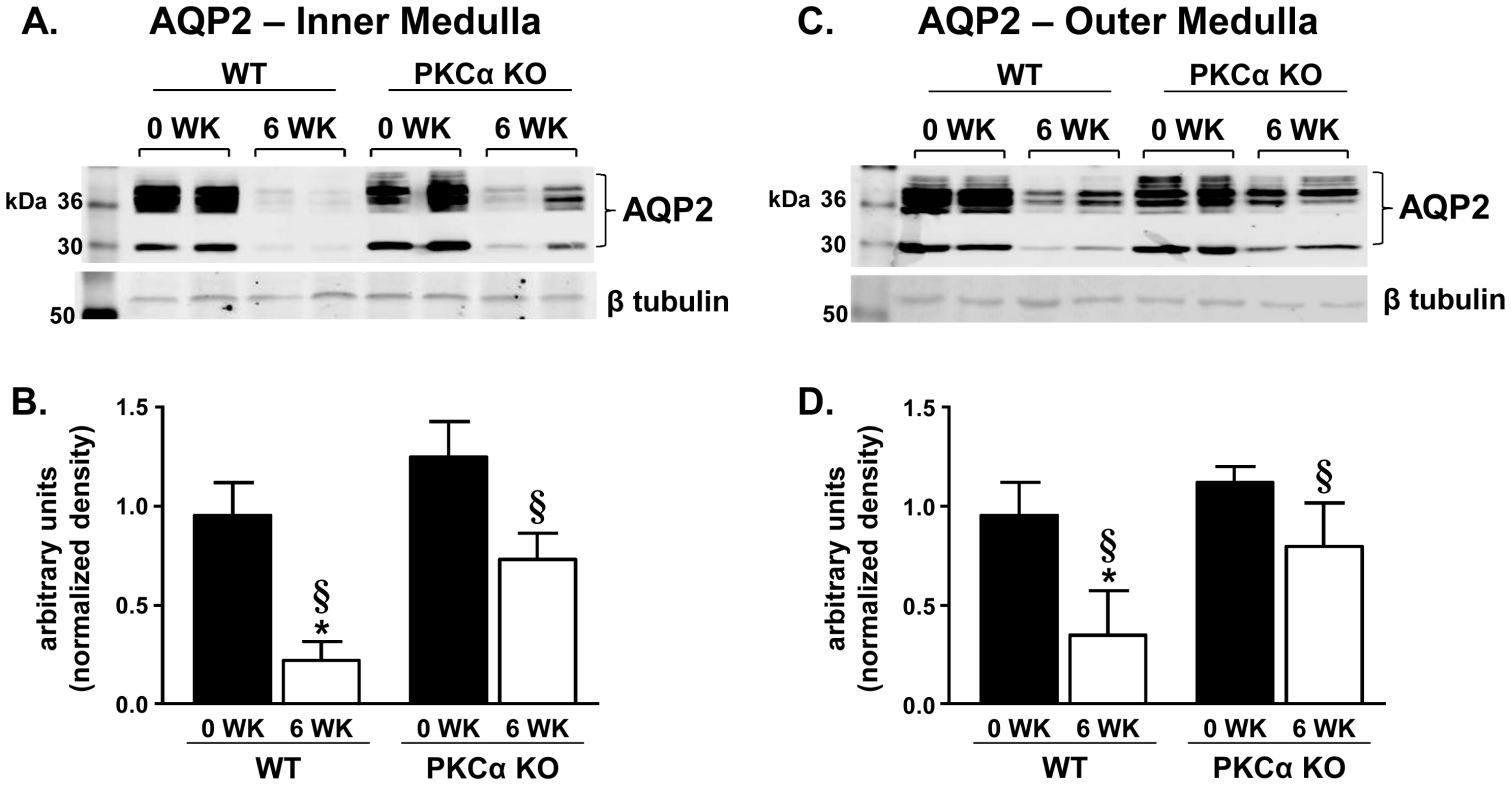
Long-term lithium treatment does not lower AQP2 expression as extensively in PKCα KO mice. IM and OM tissues collected from lithium-fed WT and PKCα KO mice were subjected to Western blot analysis and probed for AQP2. A) Representative blots showing both nonglycosylated and glycosylated AQP2 (bracketed) in the IM and the corresponding loading control, β tubulin. Each lane represents one animal. B) Combined densitometry of all forms of AQP2 in the IM normalized to β tubulin. C) A representative blot where each lane represents OM tissue from one animal probed for AQP2 (bracketed) and β tubulin. D) Combined densitometry of all forms of AQP2 in the OM normalized to β tubulin. Data are presented as mean ± SEM where *  =  p<0.05 vs. WT day 0 and §  =  p<0.05 vs. PKCα KO day 0 is deemed significant. *n = 12*.

**Figure 5:**
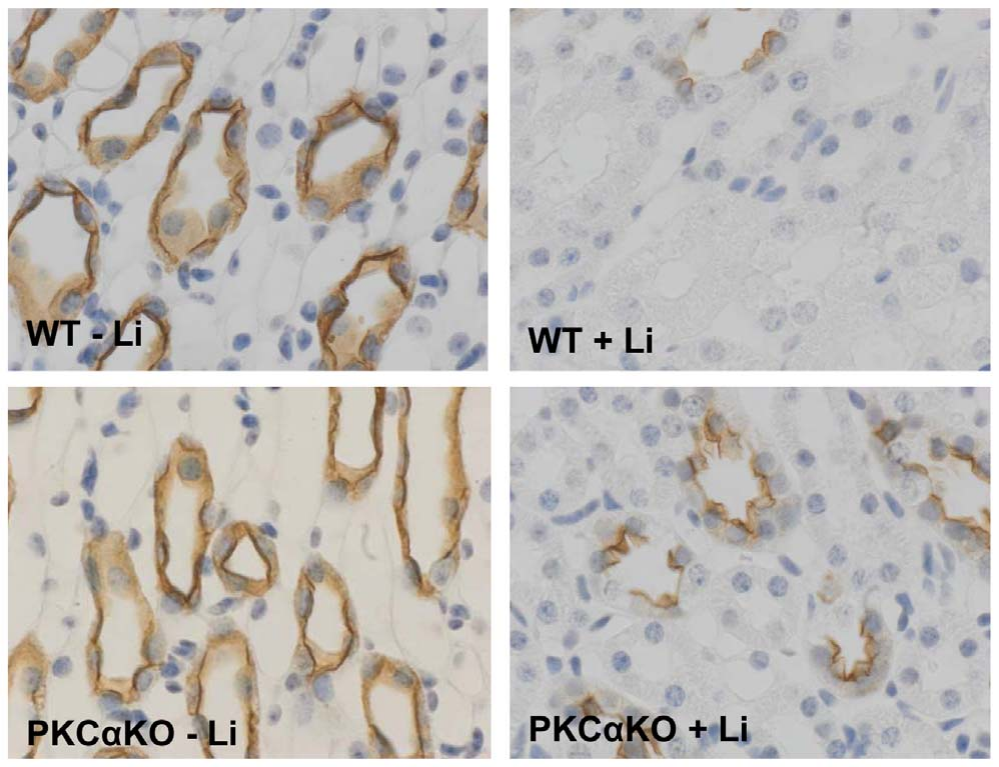
AQP2 localization to the apical membrane is increased in lithium-treated PKCα KO mice. Each panel depicts representative immunohistochemistry images (40X) of IM tissue for stained for AQP2 localization. *n = 6*.

Inner medullas collected from untreated WT and PKCα KO mice had comparable expression levels of AQP2 when quantified by Western blot analysis as expected [18] (Figure 4A). After 6 weeks of lithium treatment, AQP2 expression in the inner medulla was almost abolished in the WT mice whereas AQP2 levels were decreased 40% in treated PKCα KO mice (Figure 4B). In outer medullary tissues, AQP2 abundance was decreased in response to lithium treatment in WT mice; however, lithium only reduced AQP2 expression by 30% in PKCα KO mice (Figure 4D). Because PKCα reportedly plays a role in vasopressin-induced AQP2 trafficking [32], we wanted to determine if the preservation of AQP2 expression in lithium-treated PKCα KO was ineffective due to inability of the channel to reach the apical membrane. AQP2 was diffusely localized in the cytosol of inner medullary collecting duct cells in both untreated WT and PKCα KO mice (Figure 5). We observed that AQP2 localization was primarily apical in lithium-treated PKCα KO mice (Figure 5).

**Figure 6:**
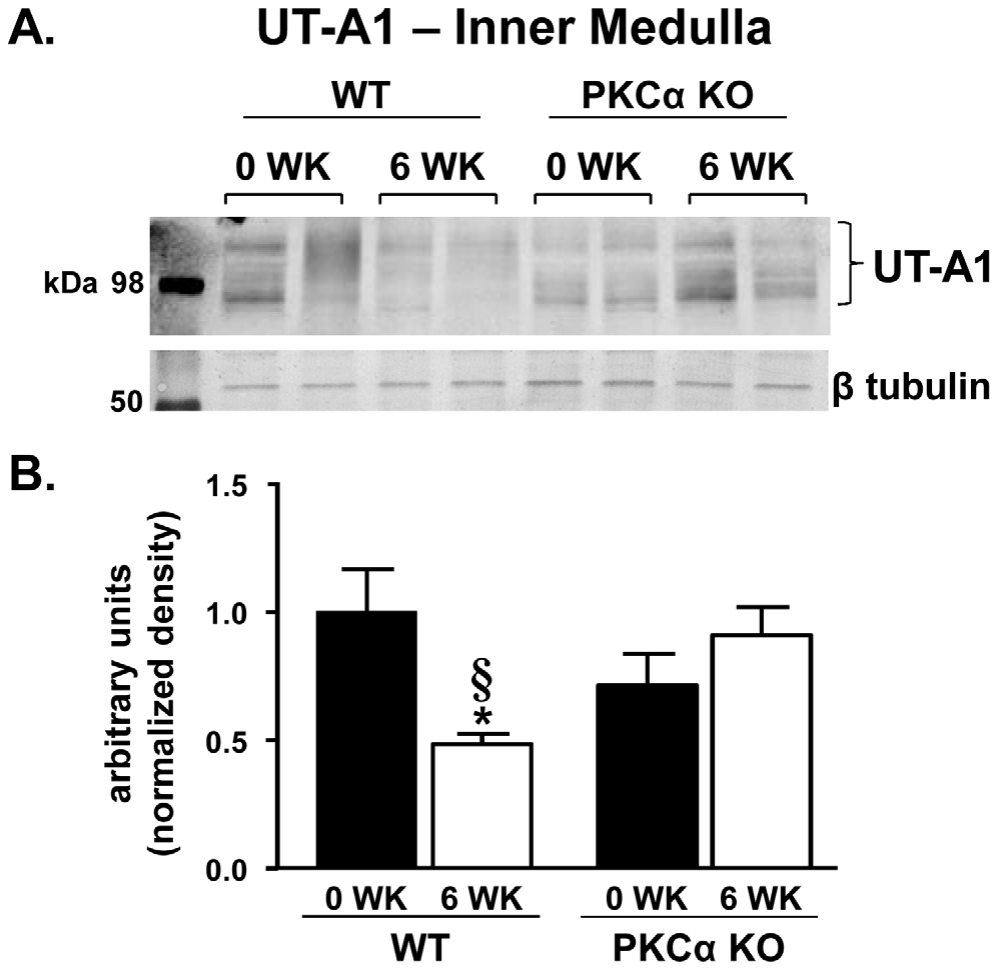
UT-A1 expression is not altered in long-term lithium treatment PKCα KO mice. IM tissue collected from lithium-fed WT and PKCα KO mice was subjected to Western blot analysis and probed for UT-A1. A) Representative blots showing the multiple glycosylated forms of UT-A1 (bracketed) and corresponding loading control, β tubulin. Each lane represents one animal. B) Combined densitometry of all glycosylated forms of UT-A1 normalized to β tubulin. Data are presented as mean ± SEM where *  =  p<0.05 vs. WT day 0 and §  =  p<0.05 vs. PKCα KO day 0 is deemed significant. *n = 12*.

**Figure 7:**
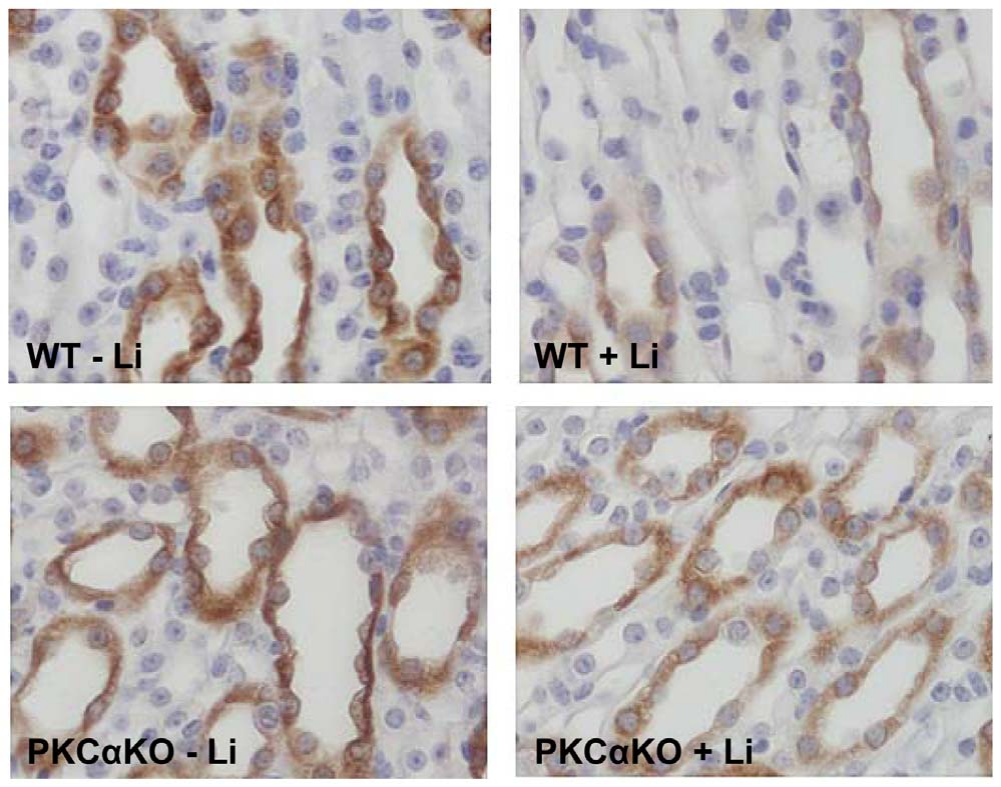
UT-A1 localization is not changed in lithium-treated PKCα KO mice. Each panel depicts representative immunohistochemistry images (40X) of IM tissue for stained for UT-A1 localization. *n = 6*.

UT-A1 expression in the inner medulla was decreased 52% in WT mice treated with lithium for 6 weeks compared to untreated WT mice (Figure 6A). Interestingly, UT-A1 expression did not decrease in PKCα KO mice treated with lithium; rather, there was a trend toward an increase in urea transporter expression although this did not reach significance (Figure 6B). Despite a decreased abundance, UT-A1 was detected in the apical region of the IMCD in lithium-treated WT mice (Figure 7). Expression and cellular localization of UT-A1 is unchanged in the PKCα KO mice treated with lithium (Figure 7).

### Electrolyte homeostasis and kidney function in long-term lithium treatment

**Figure 8:**
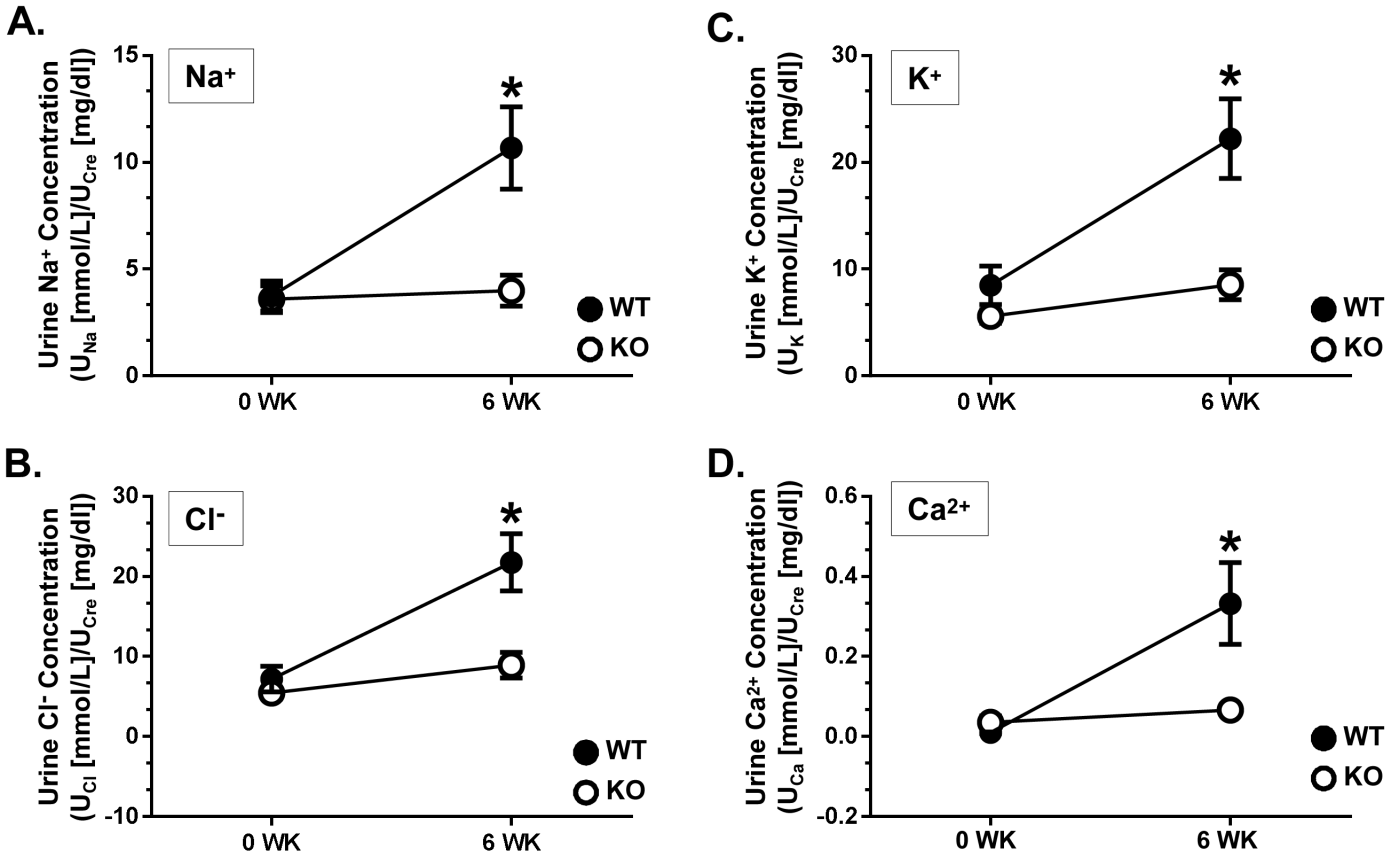
PKCα KO mice are resistant to lithium-induced natriuresis, kaliuresis and hypercalciuria. Urinary sodium (A), chloride (B), potassium (C) and calcium (D) were measured and normalized to urinary creatinine to examine difference between lithium-fed WT and PKCα KO to untreated control groups. Data are presented as mean ± SEM where *  =  p<0.05 untreated vs. 6 week-fed lithium treatment. *n = 12.*

Urinary sodium, chloride, potassium and calcium levels were measured in mice from all four experimental groups and normalized to urinary creatinine to compensate for urine volume (Figure 8). Following 6 weeks of lithium treatment, natriuresis was observed in the WT mice whereas the PKCα KO mice had no change in sodium excretion (Figure 8A). We observed a similar increase in potassium excretion in WT mice treated with lithium (Figure 8B). PKCα KO mice displayed no significant changes in potassium excretion (Figure 8C). Next we measured urinary calcium and found that lithium-treatment significantly elevated calcium levels in WT mice; however, urinary calcium levels remain unchanged in treated PKCα KO mice (Figure 8D).

## Discussion

Although lithium is an older antipsychotic, it still remains a popular treatment for bipolar disorder and its therapeutic potential for other central nervous system (CNS) diseases is also gaining favor [2]. The fraction of patients that develop lithium-induced NDI are more at risk to become dehydrated, increasing the risk of lithium toxicity [1], [33].

Water balance is maintained in the collecting duct by vasopressin regulation of AQP2 and UT-A1 through a cAMP pathway [26], [27]; however, recent studies suggest that other signaling pathways can also regulate the urine concentration mechanism [17], [19], [20], [34]-[36]. Lithium impairment of cAMP production [6]–[8] led to our exploration of a cAMP-independent pathway involving PKCα, which has been implicated in regulating urine concentration [16]–[18].

Our results demonstrate that in the absence of PKCα, mice are protected from lithium-induced NDI from the onset of treatment and, as the regimen progresses; the polyuria is not as severe. This may seem counterintuitive given that other studies have shown that PKCα KO mice have a higher flow rate and lower urine osmolality [18] and we observed that at basal state, PKCα KO mice are slightly polyuric (Table 1 and 2). In agreement with Yao et.al [18], we did not see a difference in medullary AQP2 expression between WT and PKCα KO mice (Figure 4) and there are no apparent alterations in the morphology of the inner medulla in PKCα KO mice (Figures 5 and 7). Despite the mild, basal polyuria in PKCα KO mice, these animals are able to concentrate urine after water depravation [18] demonstrating that the urine concentration mechanism is preserved. Although the mechanism of how PKCα contributes to urine concentration in the basal state remains to be determined, our studies demonstrate that when the concentration mechanism is tested by lithium treatment, the mild polyuria in the PKCα KO mice is overcome and the massive polyuria associated with NDI is attenuated in the absence of the kinase.

The presence of AQP2 in the IMCD is critical for production of concentrated urine to maintain fluid homeostasis [27]. Consequently, protein abundance of AQP2 is increased as a regulatory mechanism to long periods of water deprivation and/or high circulating levels of vasopressin [37]. Several animal models of acquired NDI including hypokalemia, hypercalcemia, ureteral obstruction, chloroquine and lithium nephrotoxicity have decreased AQP2 expression in the inner medulla [23], [38]–[40]; hence the need to understand the transcriptional regulation of the *AQP2* gene. It is generally accepted that the cAMP-mediated regulation of *AQP2* gene transcription is mediated by CREB binding to a documented functional CRE element [41]. Although it has been speculated that lithium reduced AQP2 expression by dampening cAMP synthesis, studies by Li et al., showed that lithium reduction of AQP2 mRNA level was independent of cAMP and vasopressin-stimulated PKA phosphorylation of CREB still occurred [20]. Our study found that ablation of PKCα prevented the reduction of AQP2 abundance commonly observed in lithium-treated control animals. The mechanism behind PKCα suppression of AQP2 is unknown. It is possible that in the absence of PKCα, Gi-mediated inhibition of cAMP generation does not occur thus allowing cAMP to remain in the IMCD cell to stimulate CREB-mediated transcription of the *AQP2* gene. Using systems biology-based approaches, Yu et al., identified several putative transcriptional regulator families and binding elements other than CREB and CRE that may regulate *AQP2* gene expression [42] suggesting that PKCα could decrease AQP2 expression by either directly phosphorylating one of these novel transcription factors or indirectly by phosphorylating one or more downstream modifying proteins. While detailed mechanisms of down regulation of AQP2 by lithium and other causes of acquired-NDI remain unexplained, our findings support a role for PKCα in this process.

Unlike AQP2, UT-A1 transcription is not mediated by cAMP but rather through the interaction between the tonicity-responsive enhancer (TonE) and the corresponding transcription factor, TonEBP [26]. Our results demonstrate that UT-A1 expression is not altered by lithium in PKCα KO mice. Preservation of the urea transporter in the absence of PKCα likely occurs through the persevered tonicity of the medullary interstitium in lithium-treated PKCα KO mice. For instance, in long-term cyclosporin A administration, another model of acquired-NDI, downregulation of TonEBP was secondary to the reduced tonicity of medullary interstitium [43]. We also observed an ensuing kaliuresis in lithium-treated WT mice. Potassium depletion reduces the abundance of TonEBP [44], possibly explaining why abundance of UT-A1 is not altered in long-term lithium-treated PKCα KO mice that are not potassium wasting. In addition to CRE, TonE is also present in the *AQP2* gene [45] which could contribute to the cAMP independent reduction of AQP2 mRNA by lithium [20].

In addition to persevering AQP2 expression, we found that in the absence of PKCα, AQP2 expression was highly localized to the apical membrane of the IMCD following long-term lithium treatment. Studies indicate that the inhibitory action of PKC on vasopressin-stimulated water permeability is due to PKC-initiated endocytosis of AQP2 [46]. Although PKC does not directly interact with AQP2 [32], activation of PKC leads to the short-chain ubiquitination of AQP2 resulting in lysosomal degradation of the channel [16]. Reports show that the PKCα isoform is involved in α-tubulin assembly in renal cells [47] and microtubule-dependent trafficking of AQP2 regulates intracellular localization following internalization; however, microtubules are not involved in the exocytic transport of the channel [48]. In fact, overexpression of a constitutively active PKCα construct in IMCD cells prevented translocation of AQP2 to the plasma membrane and resulted in AQP2 distribution throughout the cytoplasm [32]. Our data support these collective findings and indicate that in the absence of PKCα, AQP2 remains at the apical membrane despite a lithium-mediated reduction in cAMP concentration.

Recently Zhang et al., discovered that lithium-induced natriuresis and kaliuresis is not as severe in mice lacking the P2Y_2_ receptor [13]. We found that in the absence of PKCα, the onset of natriuresis and kaliuresis with lithium treatment was blocked. Given that the creatinine clearance and the osmolality:protein ratio was unchanged in either WT or PKCα KO mice in response to lithium, it is unlikely that an alteration in glomerular filtration or kidney injury is contributing to the observed natriuretic responses. The determination of any altered response of sodium and potassium transporters along the nephron is beyond the scope of this manuscript but will be intriguing for future studies. Regardless, our current findings suggest that the downstream target of purinergic signaling, PKCα, is an important regulator in water and solute absorption.

We also observed that lithium-treated PKCα KO mice did not display the hypercalciuria observed in treated WT mice. Polyuria is often clinically correlated with hypercalcemia and resulting hypercalciuria in several fluid balance disorders including the chronic administration of lithium [49], [50]. Increased urinary calcium is sensed by the calcium-sensing receptor (CaSR), located in the apical membrane of the collecting duct. Studies suggest that activation of collecting duct CaSR by elevated urinary calcium reduces the expression of AQP2 resulting in decreased water reabsorption [51]. Bustamante et al. demonstrated that cortical collecting duct (CCD) cells chronically exposed to extracellular calcium reduced both AQP2 mRNA and protein levels while CaSR gene silencing counteracted this effect [52]. AQP2 was also reduced in the inner medulla of animal models of hypercalciuria including hypercalciuric rats [53], [54] and TRPV5 null mice [55]. Furthermore, hypercalciuria is associated with elevated levels of urinary AQP2 in enuretic children [56]. PKCα, one of the conventional PKC isoforms that requires calcium for activation, is stimulated in response to extracellular calcium treatment in CCD cells [57]. These reports combined with our findings suggest an interesting interaction between PKCα, and the known association of hypercalciuria with decreased AQP2 expression and CaSR activation.

In conclusion, we discovered that the development of lithium-mediated NDI is not as severe in the absence of PKCα. Although lithium remains the drug of choice for treating psychiatric disorders including bipolar disorder, many patients experience several side effects including acquired-NDI [1]. Our findings indicate that adding a PKC inhibitor to a prescribed lithium regimen will prevent deleterious renal side effects. Treating patients simultaneously with the CNS-penetrant PKC inhibitor, tamoxifen, and lithium reduces the number of manic episodes compared to patients on lithium therapy alone [58], suggesting that pharmacological inhibition of PKC to alleviate the renal side effects of lithium will not hamper the effectiveness of lithium as an anti-psychotic but is in fact beneficial.
